# Interleukin-2-Inducible T-Cell Kinase Deficiency—New Patients, New Insight?

**DOI:** 10.3389/fimmu.2018.00979

**Published:** 2018-05-08

**Authors:** Sujal Ghosh, Ingo Drexler, Sanil Bhatia, Heiko Adler, Andrew R. Gennery, Arndt Borkhardt

**Affiliations:** ^1^Department of Pediatric Oncology, Hematology and Clinical Immunology, Medical Faculty, Center of Child and Adolescent Health, Heinrich-Heine-University, Düsseldorf, Germany; ^2^Institute for Virology, Medical Faculty, Heinrich-Heine-Universität Düsseldorf, Düsseldorf, Germany; ^3^Research Unit Lung Repair and Regeneration, Comprehensive Pneumology Center, Helmholtz Zentrum München—Deutsches Forschungszentrum für Gesundheit und Umwelt (GmbH), Munich, Germany; ^4^University Hospital Grosshadern, Ludwig-Maximilians-Universität München, Munich, Germany; ^5^German Center for Lung Research (DZL), Giessen, Germany; ^6^Paediatric Immunology and HSCT, Newcastle University and Great North Children's Hospital, Newcastle upon Tyne, United Kingdom

**Keywords:** primary immunodeficiency, combined immunodeficiency, interleukin-2-inducible T-cell kinase, Epstein–Barr virus-related malignancies, lymphoproliferative disorders

## Abstract

Patients with primary immunodeficiency can be prone to severe Epstein–Barr virus (EBV) associated immune dysregulation. Individuals with mutations in the interleukin-2-inducible T-cell kinase (*ITK*) gene experience Hodgkin and non-Hodgkin lymphoma, EBV lymphoproliferative disease, hemophagocytic lymphohistiocytosis, and dysgammaglobulinemia. In this review, we give an update on further reported patients. We believe that current clinical data advocate early definitive treatment by hematopoietic stem cell transplantation, as transplant outcome in primary immunodeficiency disorders in general has gradually improved in recent years. Furthermore, we summarize experimental data in the murine model to provide further insight of pathophysiology in ITK deficiency.

## Introduction

Epstein–Barr virus (EBV) is recognized to cause infectious mononucleosis. More than 90% of the global population carries the latent virus life-long and most individuals acquire the gammaherpesvirus by silent infection at young age. Several malignancies are associated with EBV and in the last decades patients with genetic defects of T cell signaling or cytotoxic pathway have demonstrated susceptibility to severe immune dysregulation upon EBV infection or reactivation. They usually present with fatal infectious mononucleosis, lymphoma and lymphoproliferative disease (LPD), hemophagocytic lymphohistiocytosis (HLH), and dysgammaglobulinemia (1, 2).

While many combined immunodeficiencies (e.g., defects of antigen receptor recombination *RAG1/2*) can lead to EBV immune dysregulation beside other infectious complications, there are diseases, which confer a higher propensity only of EBV associated disease. Several genes have been linked to EBV lymphoproliferation (*SH2D1A, STK4, CD27, CD70, LAT, RASGRP1, MAGT1, Coronin-1A*, and *CTPS1*) in recent years (2). Our group and others reported alterations in the interleukin-2-inducible T-cell kinase (*ITK*) gene in patients presenting with severe EBV associated dysregulation (3, 4). At least one decade earlier, murine studies had already shown that ITK is essential for various T cell functions, especially during a Th2 response. In this mini review, we update on clinical and immunological aspects in reported individuals and highlight the extensively investigated murine *itk−/−* model.

## ITK Deficiency—Clinical Presentation and Diagnosis

The first patients were reported in 2009 by our group. Two sisters from consanguineous Turkish parents presented with EBV-driven lymphoproliferative disease (3). At age of 6 years, one developed pneumocystis pneumonitis, severe candida stomatitis, cytopenia, progressive hypogammaglobulinemia, and oligoclonal polymorphic B cell lymphoproliferation.

Eighteen months later, she presented with Hodgkin lymphoma (HL), which was successfully treated with chemotherapy. However, T lymphocytes were further declining and at age of 10 years the girl succumbed to pneumocystis pneumonia. The younger sister presented with pancytopenia and severely impaired hepatic function due to EBV-associated HL. Due to rapid clinical deterioration haploidentical peripheral blood stem cell transplantation (SCT) was performed as a salvage therapy, but unfortunately the patient died due to airway obstruction during aplasia. Genome-wide linkage analysis identified *ITK*, in which the causative homozygous R335W mutation was revealed. To date, we are aware of *ITK* mutations in 17 patients originating from Greece, India, Italy, Iran, Morocco, Pakistan, Palestine, and Turkey (16 patients described in Table 1) (5–12). These patients manifested between 2.5 months and 13 years of age and presented with fever, hepatosplenomegaly, lymphadenopathy, and EBV viremia. One patient was diagnosed at birth due to family history of disease in the older brother. Thirteen patients presented either with HL or with EBV-driven B cell lymphoproliferative disease (in some cases developing to Hodgkin or large B cell lymphoma), only two showed a classical non-HL histology. In a few patients, other viral infections including CMV and VZV were seen. Given the severe immune dysregulation, at least three patients developed autoimmune phenomena and two patients developed HLH.

**Table 1:** Clinical and laboratory findings in *ITK*-deficient patients.

Origin Mutation	Patient 1 Turkey c.1003C>T: p.R335W	Patient 2 Turkey c.1003C>T: p.R335W	Patient 3 Palestine c.1764C>G: p.Y588X	Patient 4 Palestine c.1764C>G: p.Y588X	Patient 5 Palestine c.1764C>G: p.Y588X	Patient 6 Morocco c.86G>A: p.R29H	Patient 7 India c.1497delT: p.D500TfsX4	Patient 8 Iran c.468delT: p. L157Ffs*108	Patient 9 Turkey c.49C>T: p.Q17X	Patient 10 Italy/Greece Comp-het c.49C>T/c.922delG: p.Q17X A308Lfs*24	Patient 11 Turkey c.49C>T: p.Q17X	Patient 12 Turkey c.49C>T: p.Q17X	Patient 13 Turkey c.1003C>T: p.R335W	Patient 14 Turkey c.1003C>T: p.R335W	Patient 15 Pakistan c.626G>A: p.W209X	Patient 16 Pakistan c.626G>A: p.W209X
Sex	Female	Female	Female	Male	Male	Male	Female	Female	Male	Female	Female	Male	Male	Female	Male	Male
Age at diagnosis	5	6	4	5	3	11	6	13	18	5	6	2.5	7	3	4	Birth
Status	Died at age 10	Died at age 7 after HSCT	Died at age 6	Remission after Cx, age 12	a/w after HSCT, age 8	Died at age 26	Died after HSCT at age 8	Died at age 15	Unknown	a/w after HSCT age 5	Died at 8	Remission after Cx	a/w after HSCT age 8	Died at 3	a/w after HSCT age 4	a/w after HSCT age 1
Fever	+	+	+	+	+	+	+	+	+	n.a.	n.a	+	+	+	+	+
Lymphadenopathy	+	+	+	+	+	+	+	+	None	+	+	+	+	+	+	+
Hepatosplenomegaly	+	+	+	None	+	Unknown	None	+	None	+	+	+	+	+	+	None
Pulmonary involvement	+	None	None	+	+	+	+	+	Infections	None	+	None	+	+	+	+
Histology	B cell LPD Hodgkin	HL-like B cell LPD	HL	HL	HL	B cell LPD	B cell LPD, LBCL, LG	B cell LPD	None	HL	NHL	HL	LG, Burkitt	n.a.	HL-like LPD	DLBL-like LPD
Autoimmunity	None	None	None	Nephritis, thyroiditis	Thyroiditis	AIHA/ITP	None	None	None	None	None	None	None	None	None	None
HLH	None	(+)	+ (at relapse)	None	None	None	None	None	None	After HSCT	None	None	None	?	None	None
CD4+ cells	↓	↓	Normal	↓	Normal	↓	↓	↓	↓	n.i. (after Cx)	Normal	↘	↘	Normal	↓	Normal
CD8+ cells	Normal	↓	↑	↓	Normal	Normal	Normal	Normal	Normal	n.i. (after Cx)	Normal	Normal	Normal	Normal	↓	Normal
NKT cells	n.d.	↘	n.d	↘	n.d.	↘	↘	n.d.	↘	n.i. (after Cx)	n.d.	n.d.	n.d.	n.d.	n.d.	n.d.
Serology	VCA-G+, VCA-M−, EA-G+, EBNA-G−	VCA-G+, VCA-M−	VCA-G−, VCA-M−, EBNA+	VCA-G+, VCA-M−, EBNA-G−	n.d.	VCA-G+		VCA-G+	Negative	n.d.	VCA-G+, VCA-M−, EA-G−, EBNA-G−	VCA-G+, VCA-M−, EA-G−, EBNA-G−	VCA-G+, VCA-M−, EA-G−, EBNA-G+	VCA-G+, VCA-M+, EA-G+, EBNA-G+	n.d.	n.d.
Viral load at presentation	+ (n.q.)	10^3^	10^5^	10^3^	10^5^	+ (n.q.)	10^3^	10^7^	10^3^	n.d.	10^4^ CMV	10^4^	None	n.a.	10^4^	10^4^
Peak viral load	10^7^	10^4^	Unknown	Unknown	Unknown	10^6^	10^4^	10^7^	10^3^	n.d.	10^4^	10^4^	n.a.	n.a.	10^4^	10^4^

*AIHA, autoimmune hemolytic anemia; Cx, chemotherapy; HL, Hodgkin lymphoma; ITP, immune thrombocytopenia; LBCL, large B-cell lymphoma; LG, lymphomatoid granulomatosis; LPD, lymphoproliferative disease; n.d., not determined; n.q., not quantified; HLH, hemophagocytic lymphohistiocytosis; ITK, interleukin-2-inducible T-cell kinase*.

*↓, decreased; ↘, lower margin; ↑, increased*.

The number of ITK patients is too few to deduce valid statistics. However, it appears that HLH occurs less frequent in ITK deficiency than, e.g., in SLAM-associated protein (SAP) deficiency (30%) (13).

As known from other disorders with EBV predisposition, pulmonary interstitial nodules were seen in most patients. Furthermore, progressive hypogammaglobulinemia and loss of CD4+ T cells was detected, in particular naive CD45RA+ CD4+ T cells were decreased. In parallel with other EBV prone disorders (e.g., SAP deficiency), peripheral NKT cells [determined as CD3+, T cell receptor (TCR) Vbeta11+, TCR Valpha24+] were decreased in ITK-deficient patients supporting observations in transformed cell lines that NKT cells might be essential for anti EBV immunity (14). However, there is some evidence that EBV infection itself might decrease the number of NKT cells in these patients, as normal numbers of NKT cells are demonstrated in EBV-naive patients, e.g. in patients with XIAP deficiency (15). Furthermore, there are disorders with a global lack of NKT cells, in which individuals are rather susceptible to Mycobacteria, but not to EBV infection (16).

Peak EBV viremia in ITK-deficient patients was quite heterogeneous in reported patients (10^4^–10^8^ copies/μg DNA). Unfortunately, we obtained incomplete information on serological phenotype at time of manifestation to predict the time between infection and clinical exacerbation; EBV-VCA-IgM was detected in one patient only. In contrast to one of the most similar immunological disorders—SAP deficiency—there is not a single reported EBV-VCA-IgG seronegative symptomatic EBV-LPD patient highlighting the paramount importance of EBV infection and maybe specificity in the disease setting. Interestingly the spectrum of histopathological diagnosis is quite variable in reported patients. Bienemann et al analyzed seven of the 16 patients presented here. In six events, a classic mixed-cellular HL histology was shown, while the other lymphoproliferative events were rather heterogeneous (polymorphic: three events, borderline polymorphic to monomorphic blast-rich B-cell LPD: two events, HL-like B-cell proliferation: two events and large B-cell lymphoma like LPD: two events). In contrast to many immunocompromised patients (who rather demonstrate latency type III), ITK-deficient patients had predominantly EBV latency type II and presented often with nodal and extranodal manifestations simultaneously (6). One patient with ITK deficiency differs from the other patients in several points. An 18-year-old male Turkish patient suffered from recurrent progressive pulmonary infections and bronchiectasis, but no lymphoproliferative disease. He remained EBV seronegative although PCR could detect a low EBV viral load of 1,000–2,000 copies/μl (11).

## Management and Outcome

As previously demonstrated in other EBV-LPD cases, a few patients with ITK deficiency were treated with Rituximab with some improvement. IgG substitution has conferred only temporary benefit, especially to partially ameliorate immune dysregulation manifesting as lymphoproliferation and autoimmunity; corticosteroids were not helpful in the reported cases. Eight patients died between 1 and 15 years after diagnosis (mostly due to malignancies), seven within 2 years from diagnosis. Nine patients did not receive definitive treatment. Most had a fatal outcome. Six patients died due to lymphoproliferation, while only two patients remained in remission after chemotherapy for HL. However, eight patients underwent hematopoietic SCT. Two patients died after HSCT. While one of the initial patients died during aplasia with hemorrhagic acute airway obstruction after receiving haploidentical PBSCT, another patient succumbed to severe graft-versus-host disease. Recently, three more patients have been reported at two different centers (Newcastle, UK and Paris, France), which have been presented orally at the Annual Meeting of the European Society for Immunodeficiencies in Edinburgh, September 2017. All three patients were diagnosed with Hodgkin-like lymphoma or diffuse B cell lymphoma like lymphoproliferation and were subject to HSCT. Remarkably, the Paris patient was treated with five courses of Rituximab and two injections of Brentuximab to achieve clinical remission before haploidentical T replete HSCT. We can learn from those cases that immunotherapy with Rituximab or Brentuximab can lead to partial or even complete remission and at least bridge to definitive cure. We strongly suggest that each patient should be carefully considered for early HSCT, once the diagnosis of ITK deficiency has been established.

## Interleukin-2-Inducible T-Cell Kinase

**Figure 1:**
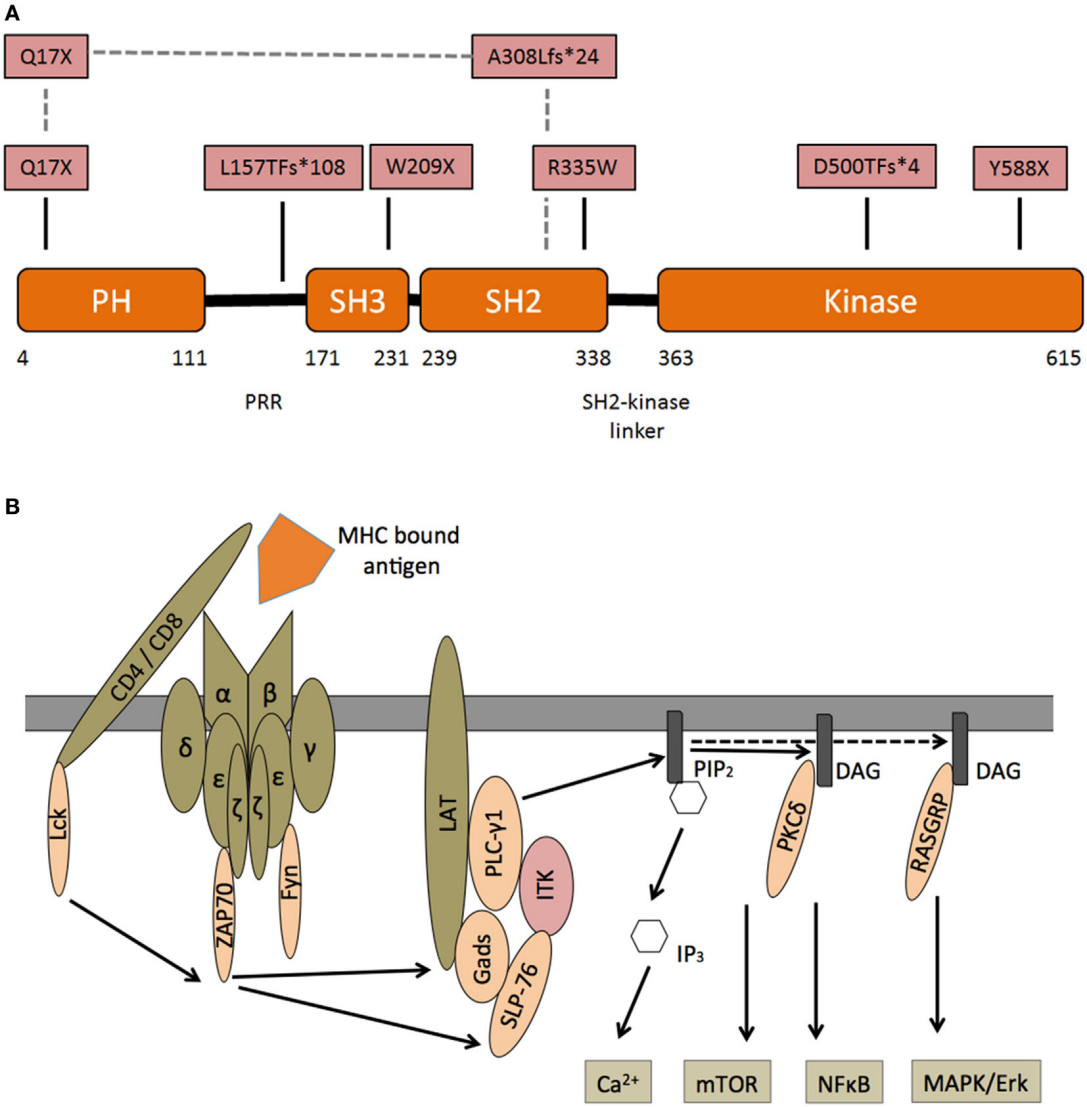
Interleukin-2-inducible T-cell kinase (ITK)—structure and signaling—**(A)** domain organization of ITK and corresponding protein mutants in patients with ITK deficiency. N-terminal pleckstrin homology (PH), Tec homology, Src homology 3 (SH3), Src homology 2 (SH2), and C-terminal catalytic kinase domain. Pattern recognition receptors. In one patient, a compound heterozygous mutation is predicted to encode Q17X and A308LfS*24 mutant. **(B)** Following engagement of the T cell receptor (TCR) with an MHC bound foreign antigen, several intracellular signals are activated. Lck is recruited and phosphorylates immunoreceptor tyrosine-based activation motifs (ITAM) at the zeta chain of the TCR. ZAP70 binds double-phosphorylated ITAM residues and phosphorylates LAT, which is recruited to the TCR complex. Phosphorylated LAT recruits SLP76, which together with Itk, activates PLCγ1. Subsequently phosphatidylinositol 4,5-bisphosphate (PIP2) is catalyzed into inositol 1,4,5-trisphosphate (IP3), which leads to intracellular calcium release and diacylglycerol (DAG). DAG itself can recruit PKCδ and RASGRP, which induce the NFκB and MAPK/ERK pathways.

Interleukin-2-inducible T-cell kinase is one of five mammal TEC family kinases. All five proteins are involved in lymphocyte signaling and development (17). Years before the first patient with ITK deficiency was diagnosed, *ITK*-*SYK* translocations were found in individuals with T cell lymphoma (18). The *ITK* gene on chromosome 5q consists of 17 exons and 112 kbp, the protein (71 kDa) is formed by 620 amino acids. ITK is composed of an N-terminal pleckstrin homology (PH), a Tec homology (TH), an Src homology 3 (SH3), an Src homology 2 (SH2), and a C-terminal catalytic kinase domain (Figure 1A) (19). Upon activation of the TCR several phosphorylation events recruit ITK to the cell membrane (for details, see Figure 1B). ITK activates PLCγ1, generating inositol 1,4,5-trisphosphate (IP3), which leads to intracellular calcium release and diacylglycerol, which, *via* RASGRP and PKCδ, ultimately results in activation/induction of the NFκB, mTOR, and MAPK/ERK pathways.

Mutations were found in the kinase, SH2 and PH domain. Most patients demonstrated an autosomal-recessive trait, while in one individual a compound-heterozygous inheritance from two different ethnicities (Greek–Italian) was revealed (5). Interestingly there are corresponding mutations in residues of the “sister” Tec kinase *BTK* (known to cause X-linked agammaglobulinemia), which are homologous to the mutations observed in our patients (9). Our group transformed Herpesvirus saimiri cell lines to reveal functional impairment in corresponding ITK mutations.

The alterations did not greatly change the amount of *ITK* mRNA expression, nevertheless immunoblot investigations showed several variants of endogenous ITK. Most importantly, we analyzed calcium response with flow cytometric flux studies and revealed a highly decreased release of calcium ions into the cytosol in most patients. With regards to functional complementation our group restored TCR-mediated calcium flux in murine *itk−/−* thymocytes by means of wild type ITK transduction.

Interestingly since the publication of our last review two new EBV prone diseases have been discovered in the proximity of ITK (with respect to location in the pathway). Linker for activation of T cells (LAT) is a transmembrane adapter molecule, which is phosphorylated after TCR triggering. It contains no intrinsic enzymatic activity and couples the TCR to downstream pathways as a scaffolding protein. PLCy1 phosphorylation is highly dependent on the assembly of the LAT-SLP76 signalosome. However, the two initial reports on two kindreds with LAT deficiency show different phenotypes and ambiguous results (20, 21). One kindred presented with a typical (severe) immunodeficiency phenotype with failure to thrive and recurrent infections. The other report describes a family with infants with increased autoimmunity due to combined immunodeficiency with a higher number of residual T lymphocytes. All three siblings suffered from CMV and EBV infection before autoimmunity developed. Similar to our investigations in ITK-deficient cell lines, both of these new reports demonstrate decreased Ca2+ mobilization and other downstream signaling in LAT-deficient Jurkat cell lines (J.CaM2.5, ANJ3), and although, ITK phosphorylation of ITK, is reported to be dependent on LAT, it was not affected in J.CaM2.5. However, the same group (with the higher number of residual T lymphocytes and autoimmunity) had the opportunity to examine calcium flux in CD45RO patient lymphocytes, which was surprisingly within the range of healthy controls 21. Interestingly, all these patients had infectious (often CMV-relate) and autoimmune problems, rather than emerging lymphoproliferation. RASGRP1 is a guanine nucleotide exchange factor, which is downstream of the PLCγ1-mediated cleavage of phosphatidylinositol 4,5-bisphosphate. Mutations in RASGRP1 have also been associated with CD4 T lymphopenia, EBV-driven B cell lymphoma, and lymphoproliferative disease (22, 23).

## *Itk−/−* Murine Phenotype

The murine *itk−/−* phenotype has been investigated for more than 25 years now, 15 years before the first patients were reported. Most studies had focused on Th1 skewing especially in infectious models; recent data further suggests that *itk−/−* CTLs harbor defects in expansion, degranulation and thus cytotoxicity. In the next chapter we will briefly summarize the *itk−/−* murine phenotype.

*Itk−/−* mice show an altered development of thymocytes with elevated numbers of innate single positive CD8+ (CD8SP) cells. These thymocytes parallel antigen-experienced T cells with a CD122+ CD44hiCXCR3+ phenotype and increased production of Eomesodermin and IFNγ, if stimulated. Similarly splenocytes (having decreased CD4 and CD8 expression in total) resemble a more differentiated phenotype (CD44+) mirroring peripheral CD8 cells of ITK-deficient patients (24–29). NKT cells have an impaired development, are dysfunctional and have a decreased survival in the periphery (30). Most studies, addressing the Th1 and Th2 paradigm suggest that ITK plays a role in a correct Th2 response (19). Upon TCR stimulation, itk−/− T cells have an impaired proliferation, less intracellular calcium release and a reduced production of effector cytokines (31).

Few epidemiological studies have observed asthma predisposition and variants in the *ITK* gene (32, 33). Several papers investigated the T lymphocytes dependent airway hyporesponsiveness in *itk−/−* mice. Pathophysiology of asthma usually involves pulmonary infiltration of Th2 cells. Due to an impaired Th2 response *itk−/−* mice show a reduced airway inflammation upon challenge with allergens (32, 34, 35). One group tried to administer an ITK inhibitor as a pharmacologic agent to suppress inflammation in already ovalbumin-induced hyperresponsive airways. Paradoxically, inhibition of ITK induced lymphoid hyperplasia, an observation they attributed to impaired cell death in the absence of cell death (32). Two studies have further focused on the impaired cell death in *itk−/−* mice, which might be at least one explanation for the lymphoproliferation seen in the patients. One study found reduced activation-induced cell death, evidenced by defective FasL upregulation upon activation and elevated T cell proliferation (36).

In recent years, Th17, Treg, and Th9 differentiation have been extensively addressed as well (37–39).

Infections show the impact of ITK on T cell differentiation and T cell effector function. In one of the first studies *itk−/−* mice on a BALB/c background failed to generate the usual Th2 response upon infection with *Leishmania major*, but rather mounted a Th1 dependent IFNγ response, which cleared the infection (31). In further studies *itk−/−* mice showed decreased granuloma formation after challenge with *Schistosoma mansoni* eggs or the nematode *Nippostrongylus brasiliensis*. Both helminths usually induce a Th2 response (31, 40). Upon *S. mansoni* infection compared to WT the size of granuloma and draining lymph nodes was significantly decreased and production of the Th2 cytokines IL-4, IL-5 and IL-10 was markedly reduced in *itk−/−* mice. Again, IFNγ levels were significantly higher suggesting Th1 skewing. If infected with *N. brasiliensis*, wild type BALB/c mice were able to fight the intestinal infection, while *itk−/−* mice showed a decrease in IL-4 and were incapable to clear the worm.

*Toxoplasma gondii*, on the other hand, promotes Th1 mediated-immunity. Although *itk−/−* mice do succumb to this infection, they are only slightly more susceptible to *T. gondii* than wild type mice (41). Serum IFN-y levels 5 days after infection and splenic IFN-y production upon stimulation after 30 days show similar values as wild type mice. Only few studies have addressed the CD8 T cell response in *itk−/−* mice. It was reported that, although *itk−/−* mice do mount protective responses to lymphocytic choriomeningitis virus Armstrong, vaccinia virus, and vesicular stomatitis virus, viral clearance is delayed, most likely due to poor activation of CD8 T lymphocytes (42, 43). Given the clinical phenotype of the reported patients, a potential role for ITK in CTL function seems highly likely. Recently, the effect of ITK on cytotoxicity and degranulation of CTLs was demonstrated. ITK-deficient CTLs showed decreased expansion and a more naïve phenotype after activation. The authors revealed that in murine *itk−/−* deficient lymphocytes, early stages of cytotoxicity were intact, while defects in degranulation were the bigger concern (44).

As far as we know there has not been any study in which an infection model of the murine gammaherpesvirus 68 (MHV-68) has been investigated in itk*−/−* mice, although murine MHV-68 infection resembles human EBV infection quite a bit. MHV-68 spreads naturally by the respiratory route and is genetically related to EBV (45). Both EBV and MHV-68 have the ability to cause infectious mononucleosis. Following intranasal inoculation the virus causes an acute infection in the lungs and remains in a latent form within B cells. Depending on CD8 T cell function, MHV-68 can further infect other splenic B cells and circulate in other organs. MHV-68 infection has already been investigated in SAP deficient mice (*Sh2d1a−/−*) leading to hypogammaglobulinemia and organ damage (46, 47). Clinically, patients with SAP deficiency have shared features with patients with ITK deficiency, hence we decided to explore the natural course of MHV-68 infection in *itk−/−* mice in some preliminary experiments. B6 and *itk−/−* mice were intranasally infected with MHV-68. There was no difference in the lytic viral tire in lungs between B6 and *itk−/−* infected mice; furthermore, there was no difference in the splenic genomic viral load between B6 and *itk−/−* mice at day 17. Clinically the mice did not behave differently. Similarly to Sh2d1a−/− mice after 3 months in total *itk−/−* mice spleens were enlarged, and we could verify a Vbeta4 expansion in all infected mice, similar to other mouse models after MHV-68 infection. Interestingly, we saw a relative decrease in CD4 cells in *itk−/−* mice; on the other hand, CD8 numbers were similar in both groups. Most importantly we saw a bigger expansion of Vbeta4 cells within in the *itk−/−* group (own preliminary results). The expansion of this clone is line with reports in Sh2d1a−/− deficient mice, and further experiments are ongoing to evaluate a potential use of this model to investigate ITK deficiency.

## Summary

Since our last review the reported patient number with ITK deficiency has nearly doubled. All patients with previous EBV infection, developed EBV-associated malignancies, like Hodgkin and non-HL and lymphoproliferative diseases, while pulmonary involvement is one of the extranodal key features. Although the number of patients is limited, a curative treatment should be considered. In settings in which an HLA-matched donor is lacking, a haploidentical donor in conjunction with advanced T-depleting and adoptive T cell transfer strategies have improved the outcome. Immunotherapy with anti-CD20 or anti-CD30 can bridge to definitive cure. EBV-negative patients (without any viremia) have not been reported yet, so we are unaware of any problems in these individuals. However, an early transplant might improve outcome. Prospective data collection on HSCT in ITK deficiency and other EBV prone primary immunodeficiencies, as CD27 or CD70 deficiency is highly warranted.

## Author Contributions

All the authors wrote the manuscript and gathered data.

## Conflict of Interest Statement

The authors declare that the research was conducted in the absence of any commercial or financial relationships that could be construed as a potential conflict of interest.
