# Corrigendum: Interleukin-2-Inducible T-Cell Kinase Deficiency—New Patients, New Insight?

**DOI:** 10.3389/fimmu.2018.02197

**Published:** 2018-10-01

**Authors:** Sujal Ghosh, Ingo Drexler, Sanil Bhatia, Heiko Adler, Andrew R. Gennery, Arndt Borkhardt

**Affiliations:** ^1^Department of Pediatric Oncology, Hematology and Clinical Immunology, Medical Faculty, Center of Child and Adolescent Health, Heinrich-Heine-University, Düsseldorf, Germany; ^2^Institute for Virology, Medical Faculty, Heinrich-Heine-Universität Düsseldorf, Düsseldorf, Germany; ^3^Research Unit Lung Repair and Regeneration, Comprehensive Pneumology Center, Helmholtz Zentrum München—Deutsches Forschungszentrum für Gesundheit und Umwelt (GmbH), Munich, Germany; ^4^University Hospital Grosshadern, Ludwig-Maximilians-Universität München, Munich, Germany; ^5^German Center for Lung Research (DZL), Giessen, Germany; ^6^Paediatric Immunology and HSCT, Newcastle University and Great North Children's Hospital, Newcastle upon Tyne, United Kingdom

**Keywords:** Epstein–Barr virus-related malignancies, combined immunodeficiency, Interleukin-2-inducible T-cell kinase, lymphoproliferative disorders, primary immunodeficiency

A mistake was made in the authorship. Heiko Adler, who contributed to the unpublished murine experiments, which are indicated in the last chapter, was unintentionally omitted from the author's list. Therefore, he should be included in the authorship. The authors apologize for the mistake. This error does not change the scientific conclusions of the article in any way.

The original article has been updated.

## Conflict of interest statement

The authors declare that the research was conducted in the absence of any commercial or financial relationships that could be construed as a potential conflict of interest.

